# Protective Effects of Pituitary Adenylate-Cyclase-Activating Polypeptide on Retinal Vasculature and Molecular Responses in a Rat Model of Moderate Glaucoma

**DOI:** 10.3390/ijms241713256

**Published:** 2023-08-26

**Authors:** Evelin Patko, Edina Szabo, Alexandra Vaczy, Dorottya Molitor, Eniko Tari, Lina Li, Adrienne Csutak, Gabor Toth, Dora Reglodi, Tamas Atlasz

**Affiliations:** 1 Department of Anatomy, ELKH-PTE PACAP Research Team, Medical School, University of Pecs, 7624 Pecs, Hungary; 2 Department of Ophthalmology, Clinical Centre, Medical School, University of Pecs, 7632 Pecs, Hungary; 3 Department of Medical Chemistry, Albert Szent-Györgyi Medical School, University of Szeged, 6720 Szeged, Hungary; 4 MTA-SZTE Biomimetic Systems Research Group, Albert Szent-Györgyi Medical School, University of Szeged, 6720 Szeged, Hungary; 5 Department of Sportbiology, Faculty of Sciences, University of Pecs, 7624 Pecs, Hungary

**Keywords:** retina, glaucoma, PACAP, neuroprotection, vasculature

## Abstract

Despite the high probability of glaucoma-related blindness, its cause is not fully understood and there is no efficient therapeutic strategy for neuroprotection. Vascular factors have been suggested to play an important role in glaucoma development and progression. Previously, we have proven the neuroprotective effects of pituitary adenylate-cyclase-activating polypeptide (PACAP) eye drops in an inducible, microbeads model in rats that is able to reproduce many clinically relevant features of human glaucoma. In the present study, we examined the potential protective effects of PACAP1-38 on the retinal vasculature and the molecular changes in hypoxia. Ocular hypertension was induced by injection of microbeads into the anterior chamber, while control rats received PBS. PACAP dissolved in vehicle (1 µg/drop) or vehicle treatment was started one day after the injections for four weeks three times a day. Retinal degeneration was assessed with optical coherence tomography (OCT), and vascular and molecular changes were assessed by immunofluorescence labeling. HIF1-α and VEGF-A protein levels were measured by Western blot. OCT images proved severe retinal degeneration in the glaucomatous group, while PACAP1-38 eye drops had a retinoprotective effect. Vascular parameters were deteriorated and molecular analysis suggested hypoxic conditions in glaucoma. PACAP treatment exerted a positive effect against these alterations. In summary, PACAP could prevent the severe damage to the retina and its vasculature induced by ocular hypertension in a microbeads model.

## 1. Introduction

Glaucoma is a common optic neuropathy characterized by the progressive loss of retinal ganglion cells (RGCs) and the degeneration of their axons that build the optic nerve. Although genetic predisposition and age are significant risk factors for the disease, increased intraocular pressure (IOP) remains the only modifiable risk factor [1,2]. Current therapies are only able to delay RGC apoptosis by lowering IOP [3]. Agents, such as alpha adrenergic receptor blockers, beta-blockers, or prostaglandins, can only facilitate the outflow of the aqueous humor (AH) or decrease fluid production [3,4]. There is a strong need for accessory therapeutic treatments that can prevent neuronal apoptosis [5,6]. Although the exact underlying pathogenesis of neuronal apoptosis in glaucoma has not been fully clarified, evidence shows that oxidative stress, glial activation, and inflammatory reactions play a role in the pathomechanism [7,8,9]. Typical hallmarks of glaucomatous retinopathy include the reduced thickness of the retinal nerve fiber layer (RNFL), greater cup to disk ratio, and characteristic visual field defects [1,10,11]. Considerable evidence shows a correlation between retinal vessel changes and reduced retinal thickness [12].

Dysregulation of blood flow with subsequent hypoxia has been suggested to have a connection to RGC death in glaucoma [13]. The “vascular theory” of glaucoma pathogenesis hypothesizes an association between optic nerve damage and retinal vasculature changes [12,14]. The retinal and optic nerve blood flow is a tightly autoregulated system, the disruption of which will lead to retinal injury [1,15,16].

Pituitary adenylate-cyclase-activating polypeptide (PACAP) is a neuropeptide with several biological functions. It has two biologically active forms: the 27 amino acid long PACAP1-27 and the longer PACAP1-38. As PACAP1-38 is the dominant form of the peptide, we applied the longer form in our experiments and refer to it simply as PACAP in the manuscript. It is the most conserved member of the secretin/glucagon/vasoactive intestinal peptide (VIP) superfamily [17,18,19]. PACAP elicits its actions through G-protein-coupled receptors, PAC1 and VPAC1/2, which can also bind VIP [19,20,21]. Soon after its discovery, it had become evident that PACAP has strong neuroprotective effects in various in vivo and in vitro models such as cerebral ischemia, Huntington’s disease, and Parkinson’s disease [22,23,24,25]. PACAP is now considered as a potent neuroprotective and cytoprotective peptide with potential therapeutic effects. In the retina, PACAP has been shown to be protective in several models of retinopathy. Our research team found that PACAP could protect against ischemia-induced changes and promote the anti-apoptotic pathways [26,27,28]. Furthermore, PACAP counteracts the damaging effects of UV light, oxidative stress, optic nerve transection, excitotoxins, hyperoxia/hypoxia, hyperglycemia, and endotoxins [17,27,28,29,30,31]. We have previously proved that PACAP is able to pass through the ocular barriers with an appropriate vehicle [32], and that PACAP, in the form of eye drops, is protective in a hypertensive glaucoma model [33]. Given the importance of vascular changes and hypoxia in glaucoma, the purpose of the present study was to further investigate the potential protective effect of PACAP eye drops on glaucomatous vasculature and on the subsequent hypoxia markers.

## 2. Results

### 2.1. Morphological Changes of the Retina

**Figure 1:**
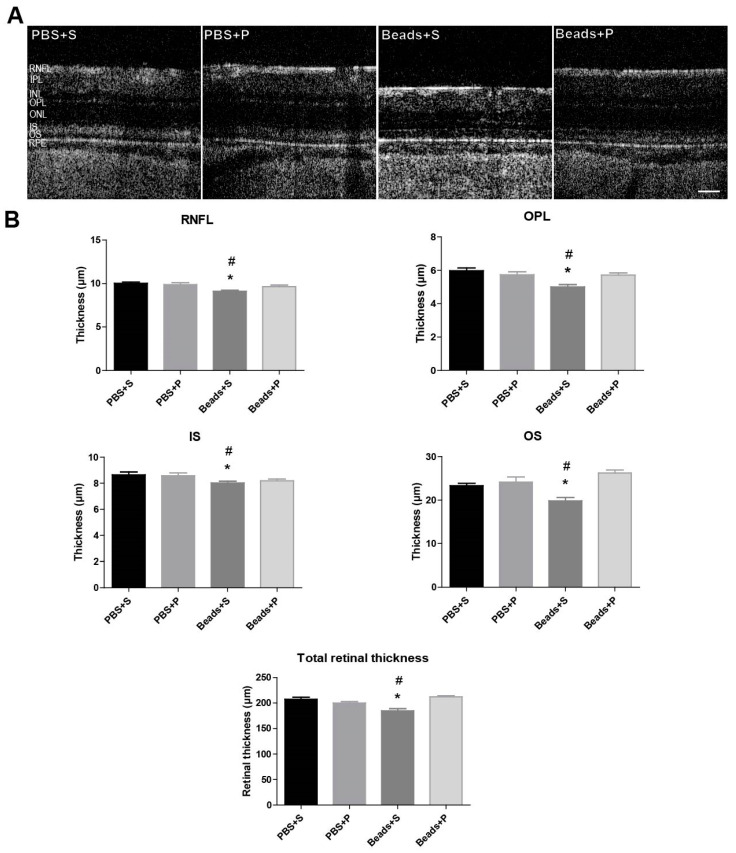
Optical coherence tomography (OCT) results. (**A**) Retinal OCT images demonstrating the normal retinal architecture in the control groups (PBS+S; PBS+P). In the glaucomatous vehicle-treated group (Beads+S), remarkable retinal changes can be observed. A significant amelioration of the retina was detected after PACAP1-38 topical administration (Beads+P). Scale bar: 50 µm. (**B**) Significant differences could be seen in the Beads+S group compared to the control groups in the following layers: RNFL, OPL, IS, OS, total retinal thickness. Values are expressed as mean ± SEM, analyzed by ANOVA and Fisher’s post hoc test. * *p* < 0.05, Beads+S vs. PBS+S; # *p* < 0.05, Beads+S vs. Beads+P; (Abbreviations: RNFL: retinal nerve fiber layer, IPL: inner plexiform layer, INL: inner nuclear layer, OPL: outer plexiform layer, ONL: outer nuclear layer, IS: inner segment, OS: outer segment, RPE: retinal pigment epithelium; PBS: phosphate-buffered saline; S: Systane; P: PACAP1-38).

Retinal morphology was analyzed in vivo during the examination period. OCT retinal scan images were made one day before the microbead injection and at the end of the examination period. PACAP administration in PBS-injected animals did not result in any alterations in the retinal layers (Figure 1A). Retinal layers in the microbeads-injected vehicle-treated animals (Beads+S) showed signs of severe retinal degeneration compared to the PBS controls (Figure 1A,B). In the Beads+S group, a significant difference was detected in the RNFL thickness (9.10 ± 0.12 µm) compared to the PBS-injected controls (10.02 ± 0.16 µm) and to the PACAP-treated microbeads-injected group (9.64 ± 0.18 µm). Also, the outer plexiform layer (OPL) thickness decreased (5.00 ± 0.14 µm) in contrast to the control groups (5.97 ± 0.16 µm) and the PACAP-treated group (5.72 ± 0.13 µm). The photoreceptors showed a notable reduction compared to the control groups both in the inner segment (IS) (8.03 ± 0.12 µm) and the outer segment (OS) (19.82 ± 0.76 µm). A remarkable decrease was observed in the total thickness of the Beads+S group (184.77 ± 4.38 µm) compared to the control groups (203.44 ± 2.51 µm). PACAP treatment led to a significant amelioration in the total retinal thickness (212.10 ± 2.36 µm) (Figure 1A,B). In summary, the microbeads-injected vehicle-treated group showed the typical signs of glaucoma with structural degeneration, while PACAP treatment could counteract the deteriorating effects of high IOP.

### 2.2. Vessel Analysis

**Figure 2:**
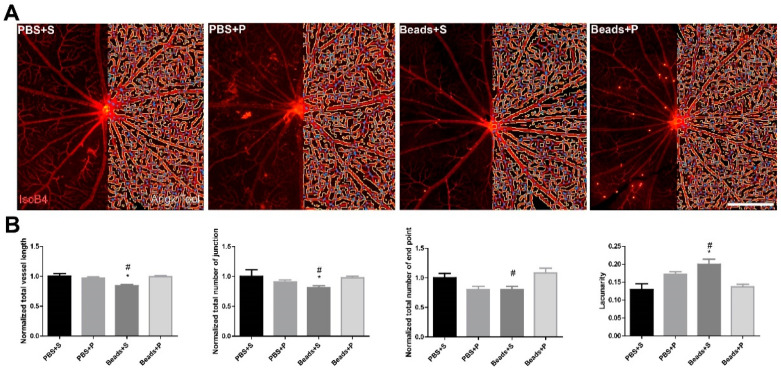
Assessment of retinal capillary network. (**A**) Whole-mount retinas were labelled with isolectin B4 and visualized to examine blood vessel morphology (**left** panel). Blood vessels were reconstructed and analyzed with AngioTool (**right** panel). Red color indicates the vessels, blue color the junction points, and yellow color the lacunarity. (**B**) Following the hypertensive conditions in the microbeads-injected vehicle-treated group (Beads+S), significant remodeling occurred, indicated by the decrease in the total vessel length, number of junctions, and end points and an increase in lacunarity, which indicates reduced vessel coverage of the retina. In the case of the PACAP1-38-treated glaucomatous group (Beads+P), these parameters remained similar to the control groups (PBS+S; PBS+P). Scale bar: 500 µm. Values are expressed as mean ± SEM, analyzed by ANOVA and Fisher’s test. * *p* < 0.05, Beads+S vs. PBS+S; # *p* < 0.05, Beads+S vs. Beads+P; (Abbreviations: PBS: phosphate-buffered saline; S: Systane; P: PACAP1-38; IsoB4: isolectin B4 label).

The PBS-treated control groups did not show any remarkable vascular differences in either the vehicle-treated (PBS+S) or the PACAP-treated (PBS+P) groups (Figure 2A). Analysis of isolectin-B4-labeled retina vessels indicated a significant 20% reduction in the total vessel length in the Beads+S group compared to the control groups (Figure 2B). A major difference (19% decrease) was found in the total number of junctions in the Beads+S group compared to the PBS+S group (Figure 2B). The number of endpoints was reduced by 20% in the Beads+S group compared to the controls (Figure 2B). The lacunarity in the vehicle-treated glaucomatous group increased by approximately 20%, indicating a severely reduced vessel coverage of the inner retina (Figure 2B). In the case of the PACAP-treated (Beads+P) group, the vessel morphology was similar to that of the control groups (Figure 2A,B).

### 2.3. Vascular Density Changes

**Figure 3:**
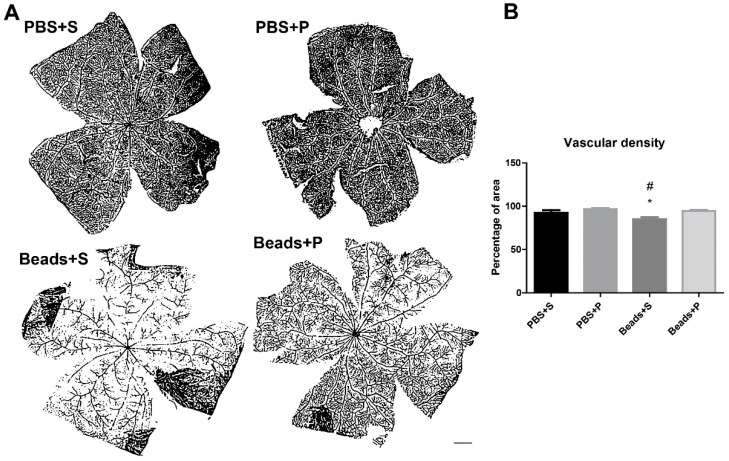
(**A**) Representative vascular images of the four groups. (**B**) Vascular density analysis suggested a significant decrease in the vessel distribution of the Beads+S group. In the PACAP1-38-treated glaucomatous group, the vascular density was similar to the control groups (PBS+S; PBS+P). Scale bar: 1000 µm. Values are expressed as mean ± SEM, analyzed by ANOVA and Fisher’s test. * *p* < 0.05, Beads+S vs. PBS+S; # *p* < 0.05, Beads+S vs. Beads+P; (Abbreviations: PBS: phosphate-buffered saline; S: Systane; P: PACAP1-38).

Microvascular densities of PBS-injected control groups (PBS+S, PBS+P) were similar (Figure 3A). However, quantitative analysis of the vascular density showed a significant reduction (20%) in the Beads+S group compared to the controls (Figure 3B). In the Beads+P group, vascular density remained similar to the controls (Figure 3A,B).

### 2.4. Immunohistochemical Changes

**Figure 4:**
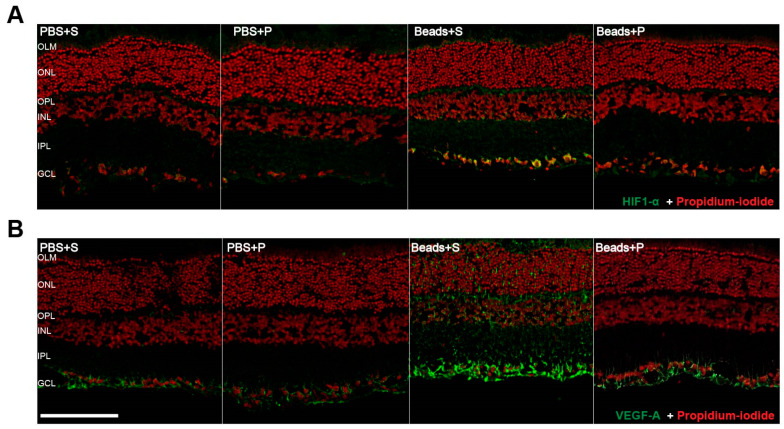
Representative vertical retinal sections (**A**,**B**) (retinal areas within 1 mm of the optic nerve) stained by HIF1-α (**A**) and VEGF-A (**B**) antibodies showing the effects of elevated IOP in the four examined groups (PBS+S, PBS+P, Beads+S, Beads+P). Increased IOP resulted in an elevation of HIF1-α (green) (**A**) and VEGF-A (green) (**B**) immunopositivity in the Beads+S group compared to the controls (PBS+S, PBS+P). We found that the increase in HIF1-α and VEGF-A expressions were counteracted by topical PACAP1-38 treatment (Beads+P). Scale bar: A, B: 100 µm. (Abbreviations: PBS: phosphate-buffered saline; S: Systane; P: PACAP1-38; HIF1-α: hypoxia-inducing factor 1 α; VEGF-A: vascular endothelial growth factor A, OLM: outer limiting membrane, ONL: outer nuclear layer, OPL: outer plexiform layer, INL: inner nuclear layer, IPL: inner plexiform layer, GCL: ganglion cell layer).

The PBS-injected groups (PBS+S; PBS+P) did not show any notable immunofluorescent changes either in retinal HIF1-α or VEGF-A expressions (Figure 4A,B). In the case of the Beads+S group, an intense level of HIF1-α immunopositivity was observed compared to the control (PBS+S; PBS+P) groups (Figure 4A,B). A higher level of immunopositivity was detected in hypoxic conditions in the GCL and in the IPL. The Beads+P group had a slightly higher HIF1-α positivity within the GCL than the controls. The VEGF-A expression was also more intense in the vehicle-treated glaucomatous group, particularly within the GCL and RNFL. The PACAP-treated glaucomatous group had a lower level of VEGF-A signal compared to the Beads+S group.

### 2.5. Protein Level Changes

**Figure 5:**
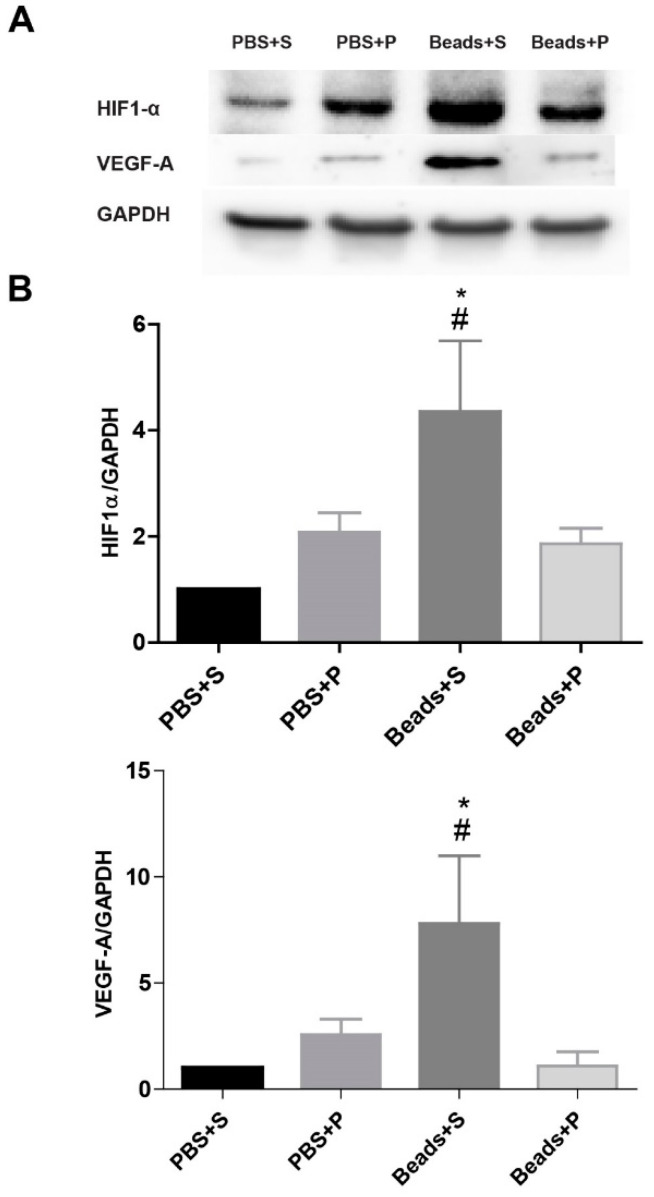
(**A**) Representative panels show the results of Western blot analysis of HIF1-α and VEGF-A from protein lysates of the four examination groups. The HIF1-α levels increased in the glaucomatous vehicle-treated (Beads+S) retina compared to controls. (**B**) Bar chart shows the relative changes of HIF1-α and VEGF-A in the four examination groups normalized to the internal control (GAPDH). Values are expressed as mean ± SEM, analyzed by ANOVA and Fisher’s test. * *p* < 0.05, Beads+S vs. PBS+S; # *p* < 0.05, Beads+S vs. Beads+P; (Abbreviations: PBS: phosphate-buffered saline; S: Systane; P: PACAP1-38, HIF1-α: hypoxia-inducible factor 1 α, VEGF-A: vascular endothelial factor-A).

We aimed to further investigate and quantitatively evaluate the protein levels of HIF1-α, and VEGF-A by immunoblotting (Figure 5A,B). In the two control groups (PBS+S; PBS+P), the HIF1-α level was low. Eight weeks after the microbead injections, the Beads+S group showed a significant elevation in the HIF1-α level compared to the control and to the Beads+P group. In the PACAP-treated glaucomatous group, similarly to the immunofluorescence results, a lower level of HIF1-α expression was detected, which was not significantly different from the control groups. For the VEGF-A protein levels, a significant increase was found in the Beads+S group compared to the control groups. After PACAP administration, we did not observe an increase in the expression of VEGF-A protein.

## 3. Discussion

In this study, we investigated the protective effects of PACAP in a rat model of glaucoma from the perspective of vascular theory. Numerous studies have previously achieved the induction of ocular hypertension (OHT) by blocking AH outflow with microbeads [34,35,36]. In our previous study, we proved that PACAP eye drops had a neuroprotective and IOP-lowering effect in the hypertensive glaucoma model [33]. Recent studies have started to focus on different aspects of the pathomechanism of glaucoma, such as vascular disruption [37]. Here, we demonstrated that PACAP is able to decrease the hypoxic conditions and preserve the retinal vasculature in the hypertensive glaucoma model.

In numerous animal studies, it has been described that PACAP and its specific receptor PAC1 are expressed in several parts of the eye, especially in the corneal endothelium and epithelium, in the ciliary body, and in the retinal ganglion and Müller cells [25]. In our previous study, we investigated the distribution of PACAP and PAC1 receptors in human eyes. We found a distribution similar to animal studies; PACAP and its receptor were present in the corneal endothelium and epithelium, in parts of the vascular layer, especially in the ciliary body, in the retinal layers, and also in the optic nerve [38]. The ability of PACAP to pass through the ocular barriers in form of eye drops and the presence of the specific receptor in the ciliary body and iris provide the background for the receptor-binding of PACAP provided in form of eye drops [32].

In vivo imaging of retinal structures has been increasingly recognized as a valuable tool in the investigation of retinal degeneration in animal models [39,40,41]. Also, recent studies on glaucoma patients have confirmed that OCT enables the detection of structural damage in the RNFL. Histologically, the loss of photoreceptors has been observed in human and primate glaucoma [42,43,44]. A previous study has suggested that the thickness between the retinal pigment epithelium (RPE) and OS is associated with visual sensitivity in glaucoma [45]. In our microbeads model, the moderate hypertension induced morphological changes in several retinal layers (total retinal thickness, RNFL, OPL, IS, OS). These changes were similar to another SD glaucoma model induced by episcleral vein occlusion [45]. The segments of the photoreceptor layer showed a significant decrease, which is in accordance with our previous study where we suggested there to be functional damage of the photoreceptors [33]. In addition, electroretinography (ERG) studies have reported the involvement of both the outer and inner retina in glaucoma, with reduced and delayed a- and b-wave amplitudes [46]. These results are in accordance with our previous functional assessment data where we found significantly decreased a- and b-waves in glaucoma [33]. As was also described earlier, we could demonstrate that the outer retina is affected along with the expected thinning of the RNFL [46]. Glaucoma also affected the whole retinal thickness [33]. A decrease of 10% was observed in the RNFL layer of the Beads+S group compared to the PBS-treated groups. In another animal model of glaucoma, Lakshmanan and co-workers (2020) found an approximately 17% decrease in this layer 8 weeks after the occlusion of the episcleral vein [45]. Our present OCT results are also in accordance with our previous study. In the present study, we could also show the thinning between the OLM-ILM and the total retinal thickness. [33]. These findings in the rat model are comparable to those in human glaucoma in terms of reduced total retinal thickness in the early stage of the disease [47,48]. The application of PACAP eye drops could protect the whole retinal morphology in glaucoma and could also preserve the inner and outer layers of the retina.

The “vascular theory” of glaucoma pathogenesis hypothesizes the association of optic nerve damage and glaucoma with the changes in retinal vasculature. Microcirculatory changes have also been observed in glaucoma patients, and disrupted ocular blood flow leads to retinal injury [14]. The perfusion of the ONH depends on three key contributors: systemic blood pressure, IOP, and autoregulatory mechanisms. The retinal ganglion cells are supported metabolically and functionally by these three factors. Fluctuation of the IOP results in vascular dysregulation, which is worse than stably reduced circulation due to increased IOP. We observed compromised vascularization in the glaucomatous group, but PACAP could prevent these changes [37,49]. Animal studies found similar changes in the retinal microvasculature in a rat magnetic bead model of ocular hypertensive glaucoma [37]. In a previous study, it was described that the retinal structure changes appeared after the decrease in the retinal blood flow in glaucoma patients. Our present results suggested the disruption of the retinal vasculature in the glaucomatous group. In the case of the PACAP eye drops, the vasculature was similar to the control groups.

The dysregulation of blood flow with subsequent hypoxia in glaucoma has been suggested to have a connection to retinal ganglion cell death [16]. Immunohistochemical studies described that HIF1-α levels were elevated in human post-mortem glaucomatous retinal tissue, which indicates hypoxic conditions [50]. Accordingly, our results demonstrated a similar change in the HIF1-α levels in retinal section. Also, this difference was supported by Western blot analysis. In the present study, we obtained results similar to Zhou and co-workers [51,52] that indicate HIF1-α was increased in the retinal tissues after IOP elevation. Elevated IOP is one of the most critical risk factors of glaucoma which can result in retinal ischemia [53]. In hypoxic conditions, HIF1-α is an important endogenous signaling molecule, contributing to physiologic changes in homeostasis [54]. A hypoxic microenvironment induces the activation of HIFs. Hypoxia-inducible factor-1 is an oxygen regulated transcription factor that controls oxygen homeostasis. In hypoxic conditions, HIF1-α regulates the activation of various genes, including glucose transporters, vascular endothelial growth factor, and other genes, which increases oxygen supply or increases metabolic adaptation to the hypoxic conditions. In the affected tissues, HIFs upregulate the production of some growth factors, mainly VEGF-A, which is produced in the eye, not only by RPE but also by ganglion cells, Müller glia, pericytes, and endothelial, glial, neural, and smooth muscle cells. VEGF-A acts on small blood vessels, inducing leakage of fluid in the retina and obliteration of capillaries, causing extra hypoxia and a further increase in VEGF-A production [55,56].

There are only few studies that have examined VEGF-A in glaucoma. VEGF-A levels were shown to be increased in the plasma of glaucoma patients when compared to healthy controls and in the aqueous humor of glaucoma patients compared to their plasma VEGF-A levels [57,58]. Despite these findings, neovascularization is not impacted in glaucoma and the exact role of VEGF-A has not been examined in the glaucomatous retina [59]. We showed the localization of VEGF-A within the retina, which was similar to that previously found, primarily localized to the RGC layer and the inner nuclear layer [59,60]. Previously, Maugeri and co-workers provided evidence that PACAP is able to decrease and inhibit HIF1-α and VEGF-A expression in a diabetic macular edema model [61]. Our present results confirmed elevated expression of HIF1-α and VEGF-A in glaucoma, and our findings suggest that PACAP is able to reduce the hypoxia-induced retinal and microvascular damage by decreasing HIF1-α and VEGF-A expression in glaucoma. Although VEGF-A is responsible for neovascularization, several studies found a lack of neovascularization in glaucoma, with an increased level of VEGF-A. This paradox question still needs to be answered.

It is well known that PACAP has anti-apoptotic, anti-inflammatory, and anti-oxidant effects, leading to neuroprotection [19]. It has been stated that PACAP eye drops can suppress the symptoms of dry eye syndrome. PACAP eye drops increase tear secretion, cAMP release, and aquaporin expression in the infraorbital lacrimal gland [62]. It has also been described that PACAP has a protective effect in hypoxic conditions in BCCAO-induced retinopathy, in diabetic macular edema, and in retinopathy of prematurity [32,61,63]. This list of retinopathies is now extended to glaucoma. Studies on animals and humans suggest the presence of dopamine receptors in the anterior segment of the eye, such as the ciliary body, as well as in the retina, and DA3 receptors play a crucial role in the AH outflow. It is well known that PACAP has a neuroprotective effect on dopaminergic cells and PACAP is able to enhance the production and exocytosis of dopamine. Thus, the protective effect of PACAP might be related to the dopaminergic system [64,65]. In summary, our study provided evidence that PACAP, in a model of glaucoma, can preserve retinal structure, decrease vascular damage, and decrease hypoxia markers. These results suggest that PACAP eye drops could be a potential future therapeutic agent in glaucoma treatment. However, further study is needed to understand the exact underlying mechanism behind the protective effect.

## 4. Materials and Methods

### 4.1. Animals

This study was performed on adult male Sprague–Dawley (SD) rats (*n* = 30) weighing 300–500 g. Animals were maintained under a 12 h light/dark cycle and fed and watered ad libitum. All procedures were undertaken in accordance with the Animal Research Review Committee of the University of Pecs, Hungary (No. BA02/2000-50/2022) and directives of the National Ethical Council for Animal Research, the European Communities Council (86/609/EEC), and ARVO Statement for the Use of Animals in Ophthalmic and Vision Research. Rats were divided randomly into four experimental groups: (i) PBS + vehicle (Systane (S)) *n* = 5; (ii) PBS + PACAP1-38 (P) *n* = 5; (iii) microbeads + vehicle (S) *n* = 10; and (iv) microbeads + PACAP1-38 *n* = 10, referred to as PBS + S; PBS + P; Beads + S; and Beads + P, respectively.

### 4.2. Induction of IOP Elevation

**Figure 6:**
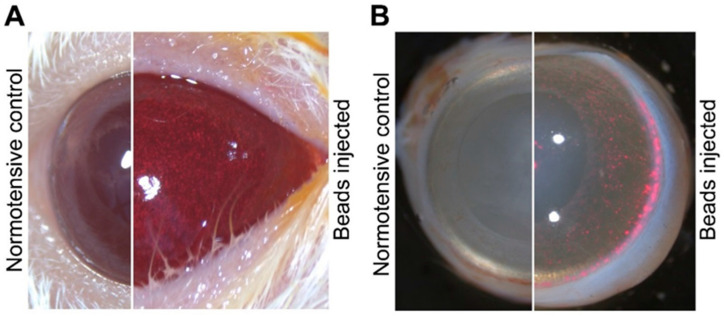
Distribution of microbeads in the anterior chamber. (**A**) Representative photograph immediately after the PBS (normotensive group) or microbeads injection. After the injection, the red microbeads start to float towards the iridocorneal angle. (**B**) Representative photograph 8 weeks after the microbeads or PBS (normotensive group) injection. Microbeads are driven into the drainage structures of the eye via the natural flow of the aqueous humor.

Intraocular pressure elevation was induced using a microbeads model, detailed previously [33]. Animals were anesthetized with intraperitoneal ketamine (90 mg/kg; Calypsol, Richter Gedeon, Budapest, Hungary) and xylazine (10 mg/kg; Sedaxylan, Dechra, Amsterdam, The Netherlands) injection. Before the microbeads injection, we applied Braunol solution (B. Braun Medical AG, Sempach, Switzerland) to prevent infections. The fluorescent (580/603 nm) polystyrene microbeads (FluoSpheres™ Polystyrene Microspheres; 10 µm Thermo Fisher Scientific; Waltham, MA, USA) (3.6 × 10^6^ beads⁄mL; 10 µL/injection) were introduced into the anterior chamber of the eyes by Hamilton syringe (33 G needle). After the injections, anti-inflammatory eye drops (Tobrex, 3 mg/mL; Alcon, Budapest, Hungary) were used to prevent inflammation and support corneal healing. The same volumes of PBS were injected into the control (normotensive) groups (Figure 6). The procedure was repeated two weeks after the first injection. During the examination period, IOP changes were recorded with a rebound tonometer (Tonolab, Icare, Vantaa, Finland).

### 4.3. Eye Drops Treatment

One day after the first injections, the eyes were treated with Systane (vehicle) solution (S) (Alcon, Budapest, Hungary) or PACAP (P) eye drops (1 µg/drop) (PACAP1-38 was synthesized at the Department of Medical Chemistry, University of Szeged, Szeged, Hungary). Rats were treated three times a day with one drop for 4 consecutive weeks.

### 4.4. Optical Coherence Tomography Examination and Morphological Analysis

Non-invasive, in vivo imaging was implemented with Optical Coherence Tomography (OCT). This technique was designed to acquire high-resolution images of the anterior chamber or the retina in real-time. Rats were anesthetized with intraperitoneal injection of ketamine (90 mg/kg; Calypsol, Richter Gedeon, Budapest, Hungary) and xylazine (10 mg/kg; Sedaxylan, Dechra, The Netherlands). Pupils were dilated with topically administered eye drops of 0.01% atropine. During the procedure, we applied artificial tear (Systane solution, Alcon, Budapest, Hungary) to protect the corneal surface. OCT imaging was performed 8 weeks after the first microbeads injections. Radial volumetric images, centered on the optic nerve, were acquired from both eyes with SD-OCT, and were analyzed and evaluated by the Bioptigen Diver program (Spectral Domain Optical Coherence Tomography (Bioptigen, Durham, NC, USA)).

### 4.5. Immunohistochemistry

Rats were sacrificed 8 weeks after the microbeads injections. Eyes (*n* = 20) were dissected in 0.1 M PBS and fixed in 4% paraformaldehyde dissolved in 0.1 M phosphate buffer (PB) for 2 h at room temperature followed by washing in 0.1 M PBS for one hour. Then, eyecups were immersed into a 10–20–30% sucrose solution and embedded in O.C.T. compound-mounting media (Tissue-Tek Cryo, Leica, Deer Park, IL, USA). Thin sections were made (15–17 μm) on gelatin-coated slides with cryostat (LeicaCM1950, BioMarker, Budapest, Hungary) and processed further for immunohistochemistry.

After rehydration with 0.1 M PBS, sections were blocked for 2 h in 5% normal donkey serum, 3% bovine serum in PBS 0.3% Triton™ X-100 (PBST) at room temperature and then incubated overnight at 4 °C with rabbit anti-HIF1-α (Sigma-Aldrich, Budapest, Hungary) diluted in 1:200 in antibody-diluting buffer or mouse anti-VEGF-A (Thermo Fisher Scientific, Waltham, MA, USA) diluted in 1:200 in antibody-diluting buffer. Immunoreactivity was detected with Alexa Fluor-488, donkey anti-mouse (Jackson Immuno Research, Cambridgeshire, UK) and Alexa Fluor-488, donkey anti-rabbit (Jackson Immuno Research, Cambridgeshire, UK) diluted 1:800 in PBST.

After, the secondary antibody sections were washed in 0.1 M PBS for one hour. Cell nuclei were stained with propidium iodine (PI). Glass slides were mounted with Fluoroshield (Sigma-Aldrich, Budapest, Hungary). Microphotographs were made with a Nikon Eclipse Ti2-E microscope with Nikon C2 confocal detector.

### 4.6. Retinal Whole-Mounts

Animals were sacrificed 8 weeks after the microbeads injections. Eyes (*n* = 24) were dissected in 0.1 M PBS and fixed in 4% paraformaldehyde dissolved in 0.1 M PB for 2 h at room temperature followed by washing 0.1 M PBS for one hour. After the washing steps, we removed the retina from the eyecup and made four small cuts. To stain the retinal vasculature, we placed the retinas in a well plate and added 500 µL of fluoresceinated isolectin solution (Isolectin GS-IB4 from Grifonia simplicifolia, Alexa Fluor568 conjugate; Thermo Fischer Scientific, Waltham, MA, USA). After an overnight rocking incubation in lectin solution at room temperature, retinas were rinsed 6 times in PBS. The labeled, isolated retinas were placed and unfolded on a glass slide. To avoid later bleaching of the fluorophores, we mounted the slides with Fluoroshield (Sigma-Aldrich, Budapest, Hungary) mounting medium. Images were made of the retinal whole-mounts with a Nikon Eclipse 80i epifluorescence microscope.

### 4.7. Vascular Analysis

For retinal blood vessel analysis, lectin-stained retina images were captured with a Nikon Eclipse 80i epifluorescence microscope (4× magnification, approximately 2500 × 2500 area, 150 µm/pixel). Images were thresholded and corrected with Adobe Photoshop CS6 (Adobe Systems, Inc., San Jose, CA, USA). Whole retinas were analyzed with the use of AngioTool. The following vessel morphological parameters were measured: total blood vessel length, total number of junctions, total number of end points, and lacunarity (the distribution of gap area surrounding the vessels). The following parameters were used in the program: blood vessel diameter (2–30 µm) and pixel intensity (0–255). Vascular density was measured using the ImageJ Vessel Analysis plugin.

### 4.8. Western Blot Analysis

For Western blot analysis, retinas were removed 8 weeks after the first injections from each separate group. Samples (*n* = 16) were processed for Western blot analysis as described earlier [31]. Protein concentrations were determined using Bradford reagents. Membranes were blocked in EveryBlot Blocking Buffer (BioRad; Hercules, CA, USA) for 5 min at room temperature and were probed at room temperature with anti-HIF1-α (1:2000; Sigma-Aldrich, Budapest, Hungary) and anti-VEGF-A (1:100; Santa Cruz Biotechnology, Dallas, TX, USA) for 1 h. Non-phosphorylated anti-GAPDH (1:20000; Cell Signaling Technology; Danvers, MA, USA) was used as internal control. Membranes were washed in Tris-buffered saline (TBS; pH = 7.5) containing 0.2% Tween. Anti-rabbit horseradish peroxidase-conjugated secondary antibody (1:3000; BioRad; Hercules; CA, USA) was diluted in EveryBlot Blocking Buffer (BioRad; Hercules; CA, USA) and the membranes were incubated for 1 h at room temperature. The antibody–antigen complexes were visualized by means of enhanced chemiluminescence. For quantification of blots, band intensities were quantified by the NIH ImageJ program (National Institutes of Health, Bethesda, MD, USA).

### 4.9. Statistical Analysis

Data are expressed as average ± standard error of the mean (SEM). Statistical comparisons were made using two-way ANOVA followed by Fischer’s post hoc analysis (OCT results; AngioTool vessel analysis; vessel density; Western blot). Differences *p* < 0.05 were considered significant.

## Data Availability

Not applicable.
